# Real-Time Bioluminescence Imaging of Mixed Mycobacterial Infections

**DOI:** 10.1371/journal.pone.0108341

**Published:** 2014-09-29

**Authors:** MiHee Chang, Katri P. Anttonen, Suat L. G. Cirillo, Kevin P. Francis, Jeffrey D. Cirillo

**Affiliations:** 1 Department of Microbial Pathogenesis and Immunology, Texas A&M Health Science Center, Bryan, Texas, United States of America; 2 PerkinElmer, Alameda, California, United States of America; Cornell University, United States of America

## Abstract

Molecular analysis of infectious processes in bacteria normally involves construction of isogenic mutants that can then be compared to wild type in an animal model. Pathogenesis and antimicrobial studies are complicated by variability between animals and the need to sacrifice individual animals at specific time points. Live animal imaging allows real-time analysis of infections without the need to sacrifice animals, allowing quantitative data to be collected at multiple time points in all organs simultaneously. However, imaging has not previously allowed simultaneous imaging of both mutant and wild type strains of mycobacteria in the same animal. We address this problem by using both firefly (*Photinus pyralis*) and click beetle (*Pyrophorus plagiophthalamus*) red luciferases, which emit distinct bioluminescent spectra, allowing simultaneous imaging of two different mycobacterial strains during infection. We also demonstrate that these same bioluminescence reporters can be used to evaluate therapeutic efficacy in real-time, greatly facilitating our ability to screen novel antibiotics as they are developed. Due to the slow growth rate of mycobacteria, novel imaging technologies are a pressing need, since they can they can impact the rate of development of new therapeutics as well as improving our understanding of virulence mechanisms and the evaluation of novel vaccine candidates.

## Introduction

Tuberculosis (TB) is a major public health problem, with over eight million new cases and 1.3 million deaths attributed to TB in 2012 [1]. Tuberculosis is a pulmonary infection that is acquired through inhalation of bacteria arising from infected individuals. The absence of a candidate with clearly superior efficacy to the current vaccine [2], an attenuated form of *Mycobacterium bovis*, bacillus Calmette-Guérin (BCG), and the unsatisfactory results of a recent anti-tuberculosis vaccine trial [3] demonstrate that additional strategies for rapid evaluation of vaccines are greatly needed. Vaccine efficacy trials would be facilitated by the ability to evaluate the challenge dose in real-time, as could be accomplished with imaging, rather than waiting the one month required for determination of bacterial load by colony forming units (cfu) with *M. tuberculosis*. Due to the difficulty of clearing *M. tuberculosis* from an infected host, successful treatment requires multiple antibiotics that must be taken for six months or more [1]. Such a long treatment time leads to patient non-compliance and has resulted in emergence of multi-drug and extensively drug resistant *M. tuberculosis* strains. The slow growth rate of *M. tuberculosis* [4], [5] makes virulence studies extremely slow in comparison to other pathogens, resulting in limited availability of information regarding mechanisms of disease establishment and progression. New tools for the study of TB in animal models are needed to facilitate development of TB prevention and treatment strategies.

Our group has been developing imaging technologies to analyze the dynamics of *M. tuberculosis* infections. We have found that tdTomato fluorescent protein labelled *M. tuberculosis* can be detected in the lungs of infected mice, but not green fluorescent protein (GFP) labelled bacteria, most likely due to the shorter emission wavelength of GFP [6], [7]. The number of bacteria required for detection by tdTomato is 10^5^ cfu, a relatively high number as compared to the infectious dose of 1–10 cfu. Interestingly, we have also used tdTomato labelled BCG infected mice to assess the efficacy of microendoscopy for detection [8], significantly improving limits of detection. In the case of *M. tuberculosis*, secreted β-lactamase BlaC can be used in reporter enzyme fluorescence (REF) to cleave fluorogenic substrates made using fluorescent dyes linked to quenchers via a β-lactam ring [9], [10]. REF allows the detection of *M. tuberculosis* in live animals without the need to genetically modify the bacteria, enabling it to be used to detect *M. tuberculosis* in clinical samples [11]. Although fluorescence systems have shown great promise, bioluminescence has the potential to allow rapid evaluation of bacterial viability and avoids the need for removal of autofluorescence normally seen in mammalian tissues.

Bacterial and eukaryotic bioluminescence systems have been used as reporters in bacteria [12]–[31]. The bacterial bioluminescence reporter systems are based on expression of the bacterial *luxCDABE* operon to produce a light signal [32]. No exogenous substrate is required, since the *luxCDE* genes code for substrate synthesis enzymes [33]. The *Photorhabdus luminescens* luciferase system [34] has been expressed from a plasmid in many different genera of bacteria, including strains of *Salmonella* that were first used to demonstrate non-invasive optical imaging in vivo [35]. Modified versions of this lux operon have been used in mycobacteria [16]. Expression of only the enzymatically active luciferase, *luxAB*, from *Vibrio harveyi*, has also been used in mycobacteria to study bacterial dissemination and antibiotic treatment efficacy, but requires delivery of an aldehyde that is poorly membrane permeable [19], [30], [31]. The eukaryotic bioluminescence systems take advantage of luciferases from insects, including the North American firefly *Photinus pyralis* and the click beetle *Pyrophorus plagiophthalamus* [24], and use a luciferin substrate that has good membrane permeability. Expression of firefly luciferase in mycobacteria has been used *in vitro* to study antibiotic resistance in different strains [12]–[16], [27], [36], [37], as well as *in vivo* [15], [18], [19], [30], [38]. Possibly the reasons that luciferases are commonly used for antimicrobial studies is their rapid loss of signal due to the requirement of ATP for light production, in contrast to fluorescent proteins that are very stable and fluorogenic probes that depend on the pharmacokinetics of the fluorescent product for signal loss [9]. The emission spectra of the insect luciferases are usually in the yellow-red range, compared to the bacterial luciferases that emit in the green to blue range [24]. Light at shorter wavelengths (e.g., blue-green) is more problematic for *in vivo* imaging, since animal tissues absorb more light at wavelengths below 600 nm [39]. Light produced by the bacteria is absorbed by the surrounding tissues and is highly attenuated [40]. Use of bioluminescence in mycobacteria has concentrated on evaluating antibiotic resistance *in vitro* [12]–[16], [27], [31], [36], [37] or *in vivo* [15], [18], [19], [30], [38].

We have developed optimized bioluminescence systems to study mycobacterial infection kinetics in real-time. We chose two luciferases, click beetle red luciferase (CBRlux) and firefly luciferase (FFlux) that have large proportions of their emission spectra in the far red wavelengths of light [40] making them good optical reporters for *in vivo* imaging. We optimized the codon usage of these genes for mycobacteria, and expressed both CBRlux and FFlux from plasmids in BCG. These bioluminescent mycobacterial strains were then used to quantify bacterial burden, both *in vitro* and in animals using non-invasive optical imaging. We show that these constructs can be used for subcutaneous detection of 10^3^ cfu *in vivo*. Moreover, the different spectra maxima of the two luciferases allow use of mixed cultures and quantitative differentiation of bacterial numbers expressing them, both *in vitro* and *in vivo*, permitting simultaneous imaging of both CBRlux and FFlux. We also demonstrate detection of these bioluminescent bacteria in the lungs of intratracheally infected mice and found that the signal is sensitive to antibiotic treatment. These observations demonstrate feasibility for simultaneous detection of multiple mycobacterial strains during infection, as well as rapid evaluation of novel intervention strategies and virulence assessment in animal models.

## Materials and Methods

### Bacterial strains and growth conditions

Strains and plasmids used in this study are listed in table 1. BCG was grown in M-OADC-Tw made with 7H9 broth (Difco, Detroit, MI) supplemented with 0.5% glycerol, 10% OADC (oleic acid dextrose complex without catalase), and 0.05% Tween 80 or Middlebrook 7H9 supplemented with 10% OADC and 15 g/liter Bacto agar (M-OADC agar) or on 7H11 selective agar (Difco) medium supplemented with 80 µg ml^−1^ hygromycin. The composition of the OADC supplement used was as described in the Difco manual [41] with the exception that it does not contain catalase. A stock solution of 1% (wt/vol) oleic acid is made in 0.2 N NaOH prior to adding 5 ml of the stock solution per 100 ml final volume of OADC supplement.

**Table 1:** Strains and Plasmids.

Strain	aGenotype	Source or Reference
**E. coli ** ***XL1 Blue***	*recA1 endA1 gyrA96 thi-1 hsdR17 supE44 relA1 lac* [F' *proAB lacI^q^ ZΔM15 Tn10 (Tet^r^)*]	Stratagene
BCG	*Mycobacterium tuberculosis* var. *bovis* bacillus Calmette-Guérin strain Pasteur	Statens Serum Institute, Copenhagen, Denmark
BCG16	BCG::pJDC134	Current study
BCG18	BCG::pJDC89	Current study
BCG26	BCG::pJDC132	Current study
BCG47	BCG::pJDC181	Current study
BCG48	BCG::pJDC182	Current study
BCG51	BCG::pJDC178	Current study
BCG52	BCG::pJDC179	Current study

Plasmid	Characteristics	
pJDC89	P_hsp60_ Hyg^r^ oriM	[42]
pJDC132	pJDC89 CBRlux	Current study
pJDC134	pJDC89 FFlux	Current study
pJDC178	pJDC89 P_L5_ optCBRlux	Current study
pJDC179	pJDC89 P_L5_ optFFlux	Current study
pJDC181	pJDC89 optCBRlux	Current study
pJDC182	pJDC89 optFFlux	Current study

a Hyg  =  hygromycin, Tet  =  tetracycline, oriM  =  mycobacterial pAL5000 origin of replication [47], [62], P_hsp60_  =  promoter from 60 kDa heat shock protein [63], P_L5_  =  promoter from the L5 mycobacteriophage [45], [64], CBRlux  =  click beetle red luciferase, FFlux  =  firefly luciferase, optCBRlux  =  click beetle red luciferase with all amino acid codons modified to the optimal codons for mycobacteria, optFFlux  =  firefly luciferase with all amino acid codons modified to be optimal for mycobacteria.

In the case of growth curves, mycobacteria were inoculated at OD_600_ of 0.02 into 96-well plates with or without different concentrations of both isoniazid (INH) plus rifampin (RIF) and incubated at 37°C. Luminescence and OD_600_ readings were taken every 2 days up to 28 post inoculation with a Mithras multimode reader (Berthold Technologies, TN). To measure luminescence of mixed cultures, strains were inoculated at an OD_600_ of 0.02 and incubated at 37°C for 10 to 14 days. The bacteria were diluted to 2×10^8^ cfu ml^−1^ and the two strains were mixed at rations of 1∶1, 10∶1, 1∶10, 100∶1 and 1∶100. Dilutions of bacterial cultures were plated on M-OADC agar and incubated at 37°C for 3–4 weeks to obtain cfu.

### Construction of luciferase expression plasmids

CBRlux and FFlux expression plasmids were constructed by cloning each luciferase gene between the *Nhe*I and *Pac*I sites of pJDC89 [42] or a derivate of pJDC89 where the P*_hsp60_* had been replaced by the P_L5_ promoter [43]–[45]. This vector uses the mycobacterial pAL5000 low-copy number origin of replication, that is stable in mycobacteria [46], [47]. CBRlux and FFlux codon sequences were optimized for expression in mycobacteria (GenScript, NJ) and the resulting genes were also inserted into pJDC89 vector via the *Nhe*I and *Pac*I sites. Codon optimized sequences have been submitted to Genbank (accession numbers: JQ031640 for CBRlux and JQ031641 for FFlux). Expression strains were constructed by transforming the plasmids into BCG by electroporation and plating the bacteria on M-OADC agar supplemented with 80 µg ml^−1^ hygromycin to select for the presence of plasmid. The presence of plasmid was confirmed in all strains by selecting multiple colonies and screening for those that produced maximal luminescence in the presence of 2 mM luciferin. In all cases, less than 10-fold variation was observed between individual colonies selected from transformants with a single construct. Luminescence was measured in white 96-well plates containing 50 µl of each bacteria suspended in culture medium and 50 µl of 2 mM luciferin added to each well and luminescence measured at 10 s post-addition and every 10 s thereafter.

### Cell lines, culture conditions and macrophage infection assays

Murine macrophage cell line J774A.1 (ATCC TIB67) was maintained in high glucose Dulbecco's Modified Eagle Medium (DMEM, Gibco) supplemented with 10% heat inactivated FBS (Gibco) and 2 mM L-glutamine at 37°C in the presence of 5% CO_2_. Cell infection assays were performed as described previously [48]. Briefly, 5×10^4^ J774A.1 cells were seeded in white 96-well plates for 20 h to allow formation of a monolayer. Mycobacteria were added at multiplicities of infection (MOI) of 100, 10, 1 and 0.1 bacteria per macrophage, incubated for 30 min to allow internalization, washed twice with pre-warmed PBS to remove extracellular bacteria, 50 µl of complete DMEM added to each well and luminescence was measured at 1, 2 and 7 d post-infection in the same manner as for bacteria in culture.

### Animal infections

Five- to seven-week old female BALB/C mice were used in all experiments. Mice were housed in groups of less than five in polycarbonate cages in a temperature, humidity and light controlled environment and provided commercial chow and tap water *ad libitum*. Mice were allowed to acclimate to the facilities for one week prior to studies. Subcutaneous infections were carried out by shaving the backs of mice and injecting dilutions of bacteria subcutaneously at specific sites in the back of each mouse. The mice were then imaged at 24 h post-infection and necropsied to determine cfu present at the site of inoculation. Alternatively, mice were infected intratracheally with 1×10^6^ bacteria, as described previously [25], [26]. Two mice per group, randomly allocated, were imaged and sacrificed for necropsy to determine thresholds of detection, correlate luminescence with cfu and compare different luminescence constructs. Antibiotic treatment was carried out with four mice per group, randomly allocated, by administration of RIF plus INH intraperitoneally daily at 10 mg kg^−1^ animal body weight. At each time point a group of animals in each treatment category were imaged, necropsied, lungs imaged and homogenized for cfu determination by plating dilutions of tissue homogenates. Animal use protocols were reviewed and approved by the Institutional Animal Care and Use Committee of Texas A&M University under AUP#2011-67.

### Imaging mycobacterial infections

Imaging was performed essentially as described previously [25], [26]. Briefly, mice were anesthetized using isoflurane and imaged using the IVIS Spectrum imaging system (PerkinElmer). Luciferin was injected intraperitoneally between 10 and 15 min prior to imaging to measure luminescence. Luciferin was injected at 150 mg/kg of animal body weight. Images were acquired with up to 5 min exposures and analysed with Living Image Software v3.1. For some experiments, spectral algorithms were used to differentiate signals from the two different luciferases. Images were acquired using different emission filters from 520 nm to 720 nm and signal quantified at each wavelength.

### Statistical Analyses

All experiments were carried out in triplicate and repeated at least two times, with similar results obtained. Data shown are for a representative experiment. The significance of the results were determined using the Student's t test for pairwise comparisons or ANOVA with the Tukey-Kramer post-hoc pairwise t test for comparisons of three or more groups. GraphPad Prism 5 software was used to facilitate statistical analyses. P<0.05 was considered significant.

## Results

### Expression of luciferases does not impact mycobacterial growth

**Figure 1:**
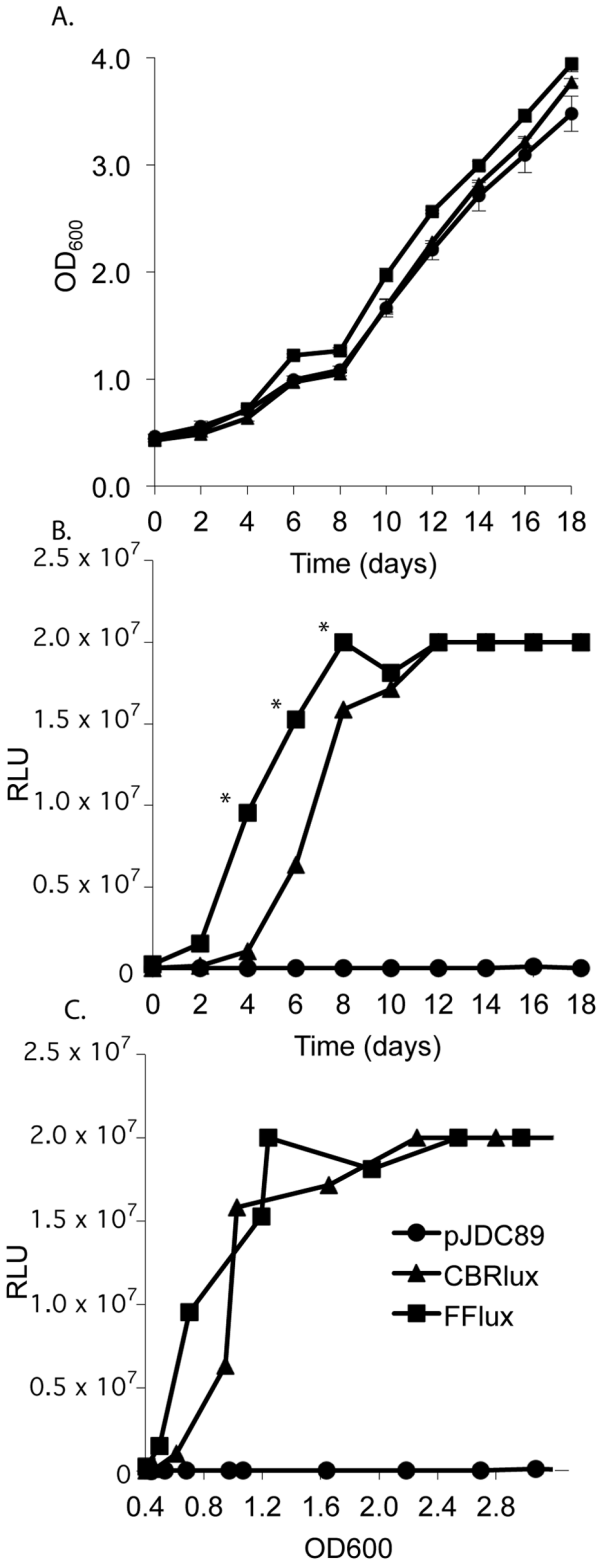
Luminescence of mycobacteria during growth in bacteriological media. Luminescent (FFlux and CBRlux) and non-luminescent (pJDC89) BCG carrying the vector backbone display similar growth rates in M-ADC-TW plus hygromycin (80 µg/ml) laboratory medium (A). Luminescence for both firefly (FFlux) and click beetle red (CBRlux) luciferase expressing BCG (BCG16 and BCG26) increases steadily over time until approximately 12 days post-inoculation (B). The correlation between luminescence and optical density of BCG cultures expressing FFlux, CBRlux or the vector (pJDC89) alone (C). Data and error bars represent the means and standard deviations, respectively, of triplicate samples. Error bars are often too small to be visible around the marker for the mean. RLU  =  relative light units. * indicate data points with P<0.05 for FFlux vs. CBRlux.

Bioluminescence from luciferases can be used to track both eukaryotic cells for cancer studies and bacterial pathogens in animal models [15], [18]–[26], [28]–[30], [35], [49]–[52]. Luciferases have great potential for tracking pathogenic mycobacteria in animal models for rapid quantitative analysis of bacterial loads in all organs during disease, vaccine efficacy studies and evaluation of novel therapeutics. Firefly luciferase (FFlux, from *P. pyralis*) and click beetle red luciferase (CBRlux, from *P. plagiophthalamus*) were cloned into mycobacterial expression vector pJDC89 under the control of two different constitutively active promoters; the P*_hsp60_* promoter or the P_L5_ promoter [43]–[45]. The resulting constructs were transformed into BCG and evaluated for growth rate and light production over 18 days (Figure 1). Luminescence correlates well with bacterial numbers during the exponential phase of growth (Figure 1C). During exponential growth, the amount of light produced increases along with bacterial numbers and plateaus at around 8 days, which is approximately the same time the cultures reach stationary phase. After reaching stationary phase, light production remains relatively constant until 20 days post inoculation. Constant light production, despite increasing bacterial numbers, is most likely a reflection of the metabolic state of stationary phase mycobacteria, since light production by coleopteran luciferases is dependent on ATP [32]. Neither luciferase had an impact on the growth rate of BCG and light production was very similar overall for both luciferases. During log phase there was a higher level of light production from FFlux as compared to CBRlux (Figure 1B), which could be due to a number of factors, including FFlux having less of a metabolic impact on mycobacteria, allowing greater light production or improved translation of the FFlux gene due to preferred codon usage. These data demonstrate that expression of both CBRlux and FFlux are well tolerated by mycobacteria and light production correlates with bacterial numbers under most conditions, suggesting that these reporters will be valuable for tracking mycobacteria during infections.

### Luciferases allow quantification of intracellular mycobacteria

**Figure 2:**
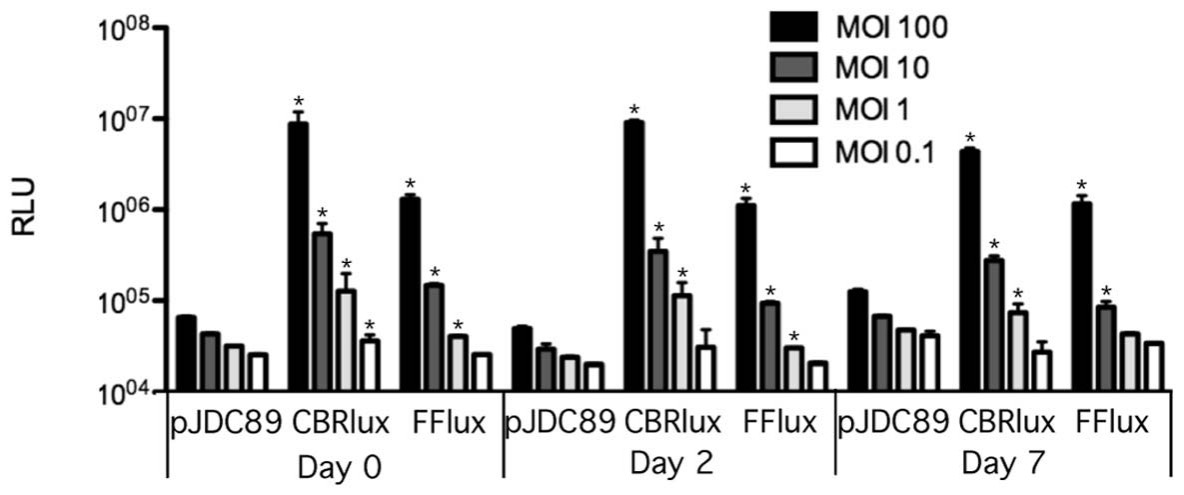
Luminescence from mycobacteria within macrophages. Luminescence detected from firefly (FFlux) and click beetle red (CBRlux) luciferase expressing BCG (BCG16 and BCG26) or BCG carrying the vector (pJDC89) alone within J774A.1 macrophages. Infections in macrophages were carried out with various multiplicities of infection (MOI) from 0.1 to 100 bacteria per cell for 30 min and washed to remove extracellular mycobacteria. Luciferin was added immediately after infection (day 0) and on days 2 and 7 post-infection ∼15 min before luminescence measurements. Data and error bars represent the means and standard deviations, respectively, of triplicate samples. RLU  =  relative light units. * indicate data points with P<0.05 for luciferase vs. pJDC89 expressing BCG at the same time point and same MOI.

Mycobacteria are primarily considered intracellular pathogens that can readily infect and replicate within macrophages [53], [54]. Since metabolic changes could impact expression of luciferase by mycobacteria, we examined whether a similar correlation with bacterial numbers was observed when the bacteria are growing within macrophages to that observed in culture. Using bacteria to cell ratios of 0.1 to 100 in murine macrophages, we found that both CBRlux and FFlux produced signal above background that correlated very well (r^2^ = 0.96 for CBRlux, r^2^ = 0.92 for FFlux) with bacterial numbers (Figure 2). This correlation is sufficient to allow accurate quantification of bacterial numbers by luminescence in the place of cfu. The level of luminescence produced was stable out to 7 days post-infection of macrophages and both CBRlux and FFlux produce similar levels of luminescence. We show that intracellular growth does not significantly impact levels of light produced by CBRlux or FFlux, allowing these reporters to be used during macrophage infection to quantify bacterial numbers present.

### Thresholds of detection for mycobacteria during mouse infection

**Figure 3:**
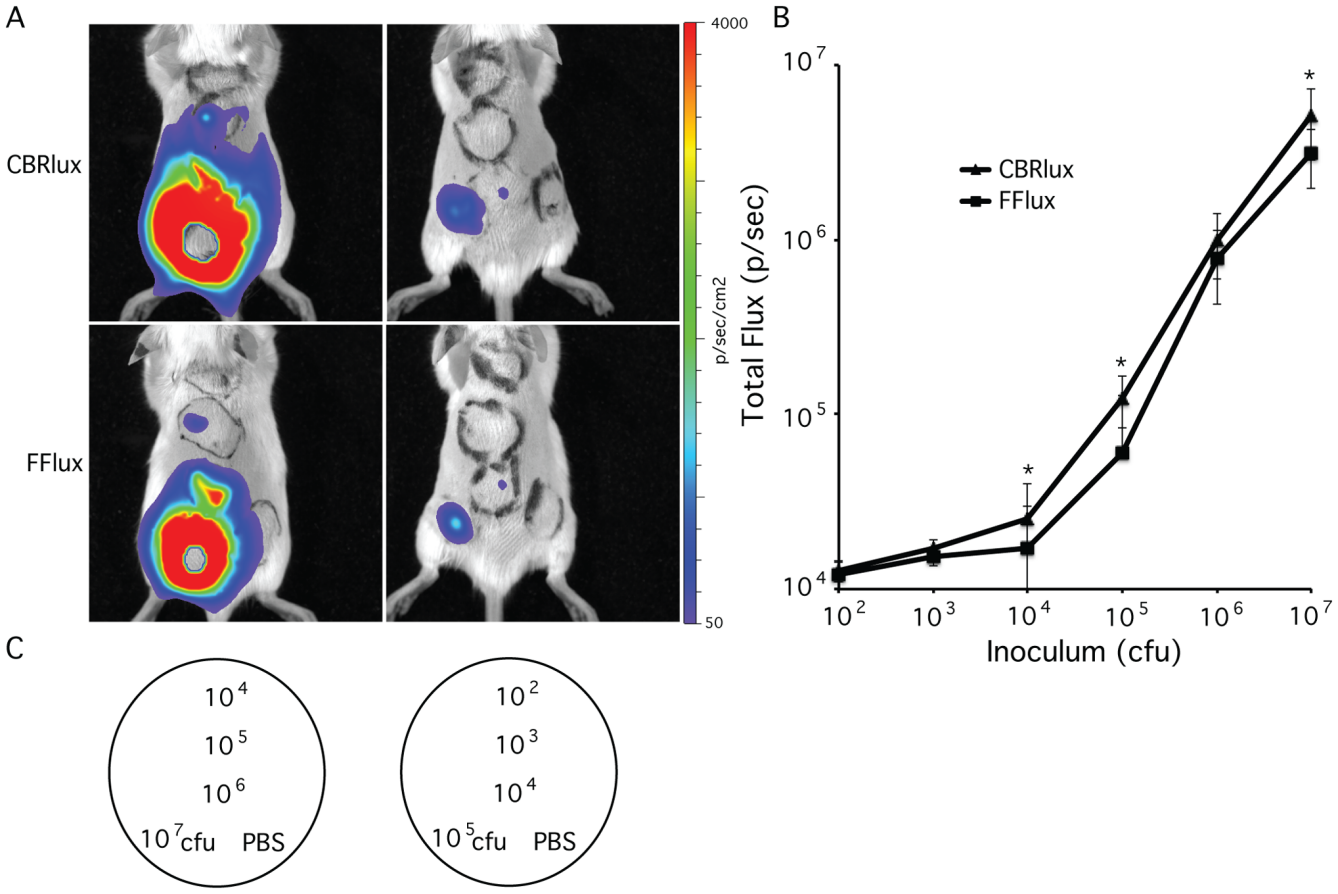
Correlation of bacterial numbers with luminescence. Whole body images of luminescence from BALB/C mice (2/group) inoculated subcutaneously with 10^2^ to 10^7^ colony forming units (cfu) of BCG expressing click beetle red (CBRlux) or firefly (FFlux) luciferase (A) at 24 h post-inoculation. After imaging, mice were sacrificed and cfu determined by plating homogenates of skin region from injection site. Bioluminescence from each inoculation site correlates well with the number of bacteria present for both CBRlux and FFlux (B). Template for injection sites using different numbers of cfu or PBS control (C). Data and error bars represent the means and standard deviations, respectively, of duplicate samples. Data are expressed as total Flux in photons per second (p/sec). * indicate data points with P<0.05 for CBRlux vs. FFlux expressing BCG at the same inoculum.

Since both CBRlux and FFlux allow quantification of intracellular mycobacteria, we examined their limits of detection for mycobacteria during infection in animals. We infected mice subcutaneously with 10^2^ to 10^7^ cfu of BCG expressing CBRlux or FFlux to determine effects of infection on light production (Figure 3). Although somewhat difficult to distinguish in whole body images due to higher numbers of bacteria saturating the dynamic range of the imager, the threshold of bacteria detected (p<0.05) using this imaging system is 10^3^ cfu for CBRlux and 10^4^ cfu for FFlux. The number of bacteria present at each site was confirmed after imaging by plating homogenized tissue for cfu and correlates well with luminescence from 10^3^ to 10^7^ cfu (Figure 3B). CBRlux displayed significantly higher luminescence than FFlux for most bacterial numbers during subcutaneous infection. This observation combined with the lower threshold of detection observed with CBRlux suggests that luminescence produced by CBRlux penetrates mammalian tissue more readily than FFlux, most likely due to the longer wavelength of luminescence produced by CBRlux as compared to FFlux [24], [40].

### Luciferases allow imaging of pulmonary infections in mice

**Figure 4:**
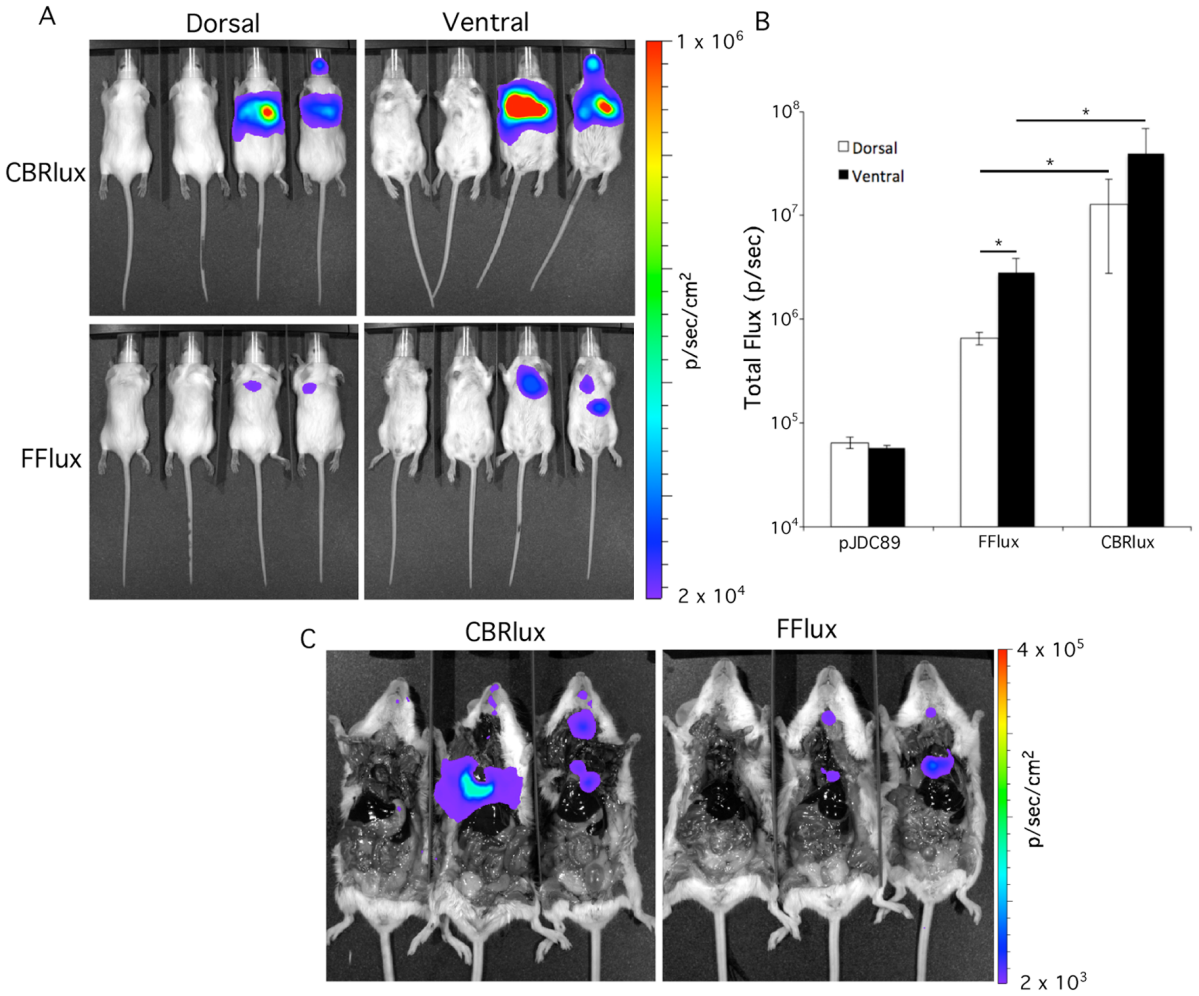
Luminescence allows imaging during pulmonary infection. Whole body dorsal and ventral images of luminescence from click beetle red (CBRlux) and firefly (FFlux) luciferase expressing BCG inoculated intratracheally into BALB/C mice (2/group) at 10^6^ cfu (A). Two vector only (pJDC89) control mice were imaged along with each set of two CBRlux or FFlux mice (vector controls on left). Data are expressed as total Flux in photons per second (p/sec). Luminescence is greater with CBRlux than with FFlux and ventral imaging is more sensitive than dorsal imaging (B). Imaging of mice post-mortem with open chest cavities demonstrates the luminescence signal originates from the lungs (C). Data and error bars represent the means and standard deviations, respectively, of duplicate samples. * indicate data points with P<0.05 between data indicated by horizontal bars.

Most mycobacterial infections occur through the respiratory route, which is a more difficult site to image optically due to the greater tissue depth than for subcutaneous infections. Although a 10^3^ cfu threshold of detection is promising to allow sensitive tracking of mycobacteria during pulmonary infections, it is unclear whether such low numbers could be detected in deeper tissues. We tested this possibility by imaging mice infected intratracheally with BCG expressing CBRlux and FFlux (Figure 4). We found that both luciferases allowed the detection of 10^6^ cfu of BCG in the lungs of mice with short exposures of 1 min resulting in 100-fold higher p/sec luminescence than background (Figure 4B). Although both dorsal and ventral images could be used to detect the presence of bacteria, ventral imaging resulted in higher luminescence for both CBRlux and FFlux. Moreover, despite bacterial numbers in the lungs for both CBRlux and FFlux strains being comparable (∼10^6^ in both cases) luminescence was nearly 10-fold higher for CBRlux than FFlux in both dorsal and ventral images. The location of the source of luminescence was confirmed post-mortem by collecting images after opening the chest cavities of the mice (Figure 4C). All of the observed luminescence originated from the lungs at 24 h post-infection. These observations indicate that ventral imaging is more sensitive for tracking mycobacterial pulmonary infections with imaging and luminescence produced by CBRlux being greater than FFlux, making CBRlux a more sensitive reporter for tracking pulmonary infections with mycobacteria.

### Spectral characteristics of CBRlux and FFlux allow simultaneous imaging

**Figure 5:**
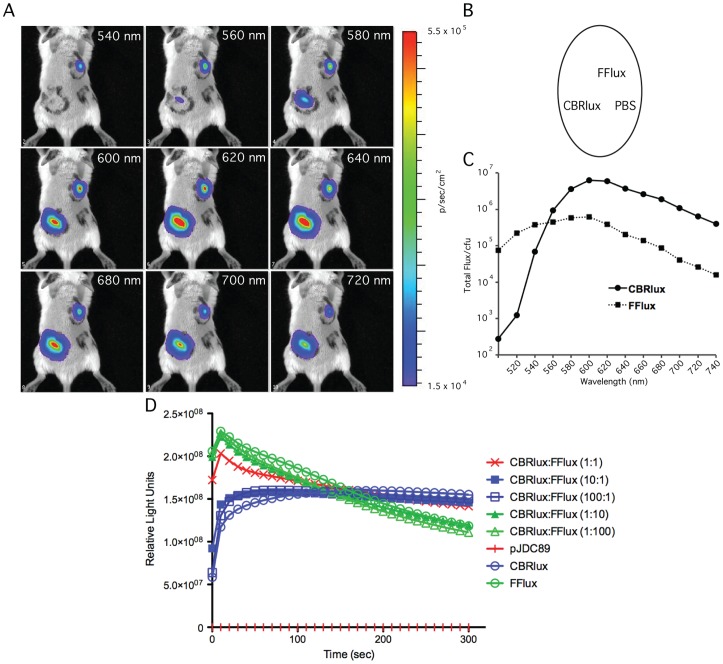
Spectral characteristics of firefly and click beetle red luciferase. Collection of whole body images of BALB/C mice (2/group) infected subcutaneously with 10^6^ cfu of BCG expressing click beetle red (CBRlux) or firefly (FFlux) luciferase at defined wavelengths between 540 nm and 720 nm demonstrates that very little luminescence is obtained from CBRlux at short wavelengths and, similarly, less is obtained for FFlux at longer wavelengths (A). Template map for subcutaneous inoculation of BCG strains and PBS control (B). Quantitation of total Flux (p/sec) at different wavelengths for areas with CBRlux of FFlux indicates that luminescence from each reporter can be spectrally separated (C). Comparing luminescence kinetics obtained with mixed cultures of 10^4^ total cfu CBRlux and FFlux expressing BCG at ratios from 1∶1 to 1∶100 shows that the kinetic curve of a mixed culture resembles that of the luciferase that is at higher numbers in the culture. Furthermore, at the similar numbers (1∶1) the kinetic curve appears to be a mix between the two curves for the luciferases. Since the kinetic curves are directly relates to the numbers of each strain present, it is likely that these luciferases compete similarly for available substrate (D). Luminescence was measured using a plate reader after injecting 50 µl of 2 mM luciferin. Measurements began 10 s after adding luciferin and were taken every 10 s up to 5 min.

Since both CBRlux and FFlux can be used to track mycobacterial infections in mice and they produce luminescence that is maximal at different wavelengths, we tested whether their signals could be separated using available algorithms for spectral unmixing. Spectral unmixing involved first capturing multiple images of the same animals, infected by both CBRlux and FFlux subcutaneously, using a range of different emission filters on a whole body optical imager (Figure 5A). We captured images of infected mice in this manner using filters from 500 to 740 nm and found that the maximum luminescence for FFlux is 540 to 600 nm and for CBRlux is 600 to 620 nm (Figure 5C). Furthermore, between 500 and 520 nm, there is almost no luminescence from CBRlux and signal obtained for FFlux is near maximal, suggesting that the spectral characteristics of these two luciferases will allow their signal to be separated and quantified.

We examined the kinetics of light production for the luciferases in mixed cultures to determine whether they competed differently for the substrate, luciferin or their presence together would interfere with levels of signal produced. We mixed cultures of CBRlux and FFlux expressing strains in 1∶1, 1∶10 and 1∶100 ratios and measured luminescence in 96-well plates (Figure 5D). After a rapid increase in light production to the maximum value within 10 s after addition of luciferin, there is a rapid decrease in the amount of luminescence for the FFlux expressing BCG strain. Light production continues to decrease over 2 minutes. Conversely, light produced by CBRlux takes slightly longer (20–30 s) to reach maximum values and stays at the maximum value for at least 2 minutes. We found that the mixing of the two luciferase emissions results in a slight increase in overall light production but that the kinetics of the light production were similar to that of the major luciferase present. However, when the two luciferases were mixed at a 1∶1 ratio, the light production resembled a combination of the two kinetic curves for each luciferase, with a lower initial maximum as compared to FFlux alone, but an extended plateau comparable to CBRlux. These results indicate that each luciferase contributes relatively equally to the total luminescence produced by mixed cultures and there is little interference between them or competition for substrate when mixed together.

### CBRlux and FFlux can be imaged simultaneously during mixed infections

**Figure 6:**
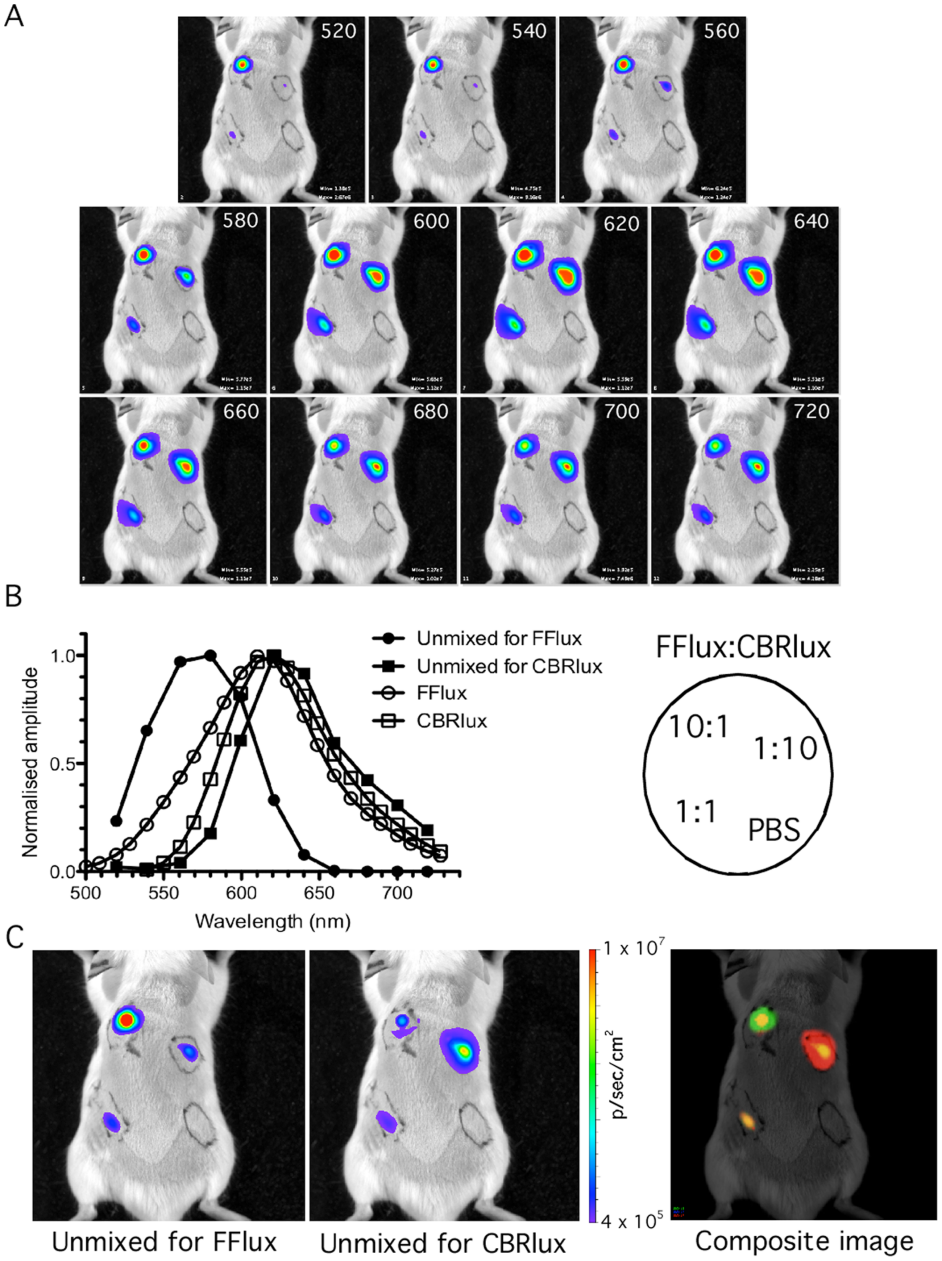
Spectral unmixing of luminescence allows quantitation of mixed bacterial infections. Whole body images were obtained from BALB/C mice (2/group) subcutaneously infected with a total of 10^7^ cfu BCG mixed at different ratios from 1∶10 to 1∶1 expressing click beetle red (CBRlux) or firefly (FFlux) luciferase at defined wavelengths from 520 nm to 720 nm demonstrates ability to quantitatively separate signal from CBRlux and FFlux in mixed infections, similar to those seen in mixed cultures (A). Normalized quantification of signal at defined wavelengths after spectral unmixing for FFlux or CBRlux (left panel) and map of subcutaneous inoculation sites on mice (right panel) for imaging (B). After unmixing signal at each injection site correlates with the number of FFlux or CBRlux bacteria present (C). The composite image (on the right) shows green for quantitative FFlux signal, red for CBRlux signal and orange at positions of co-localization of both CBRlux and FFlux signals.

The ability to independently quantify two mycobacteria expressing different luciferases during mixed infections would be extremely valuable for evaluating competition assays with mutant and wild type bacteria. We evaluated this theory by infecting mice subcutaneously with mixtures of 10∶1, 1∶1 and 1∶10 FFlux to CBRlux expressing bacteria. At 24 h post-infection, infected mice were imaged at a range of different wavelengths from 500 to 740 nm (Figure 6A) and the resulting signal at each infection site spectrally unmixed to quantify the number of FFlux and CBRlux bacteria present (Figure 6B, C). After spectral unmixing, infections with FFlux and CBRlux expressing bacteria can be differentiated and the luminescence observed at each site of infection correlated with the numbers of each bacteria present. Composite images were constructed to display the intensity of signal for each luciferase at equally infected sites (1∶1 ratio of FFlux:CBRlux) on the animal as a combination of the luminescence present at that site; whereas sites where there is a 10∶1 ratio of bacteria (10∶1 FFlux:CBRlux or 1∶10 FFlux:CBRlux), the second luciferase is observed as a smaller sector within the zone displaying luminescence for the primary luciferase present. These observations suggest that CBRlux and FFlux allow simultaneous quantification of two strains of mycobacteria, even when the two strains are in a mixed infection in mice. Two luminescence markers for imaging have numerous applications in pathogenesis studies to follow mutant and wild type strains, analysis of antimicrobial resistance when two populations are present differing in susceptibility, and evaluation of both vaccine and challenge dose in vaccine efficacy studies.

### Optimization of luciferases for spectral imaging of co-infections

**Figure 7:**
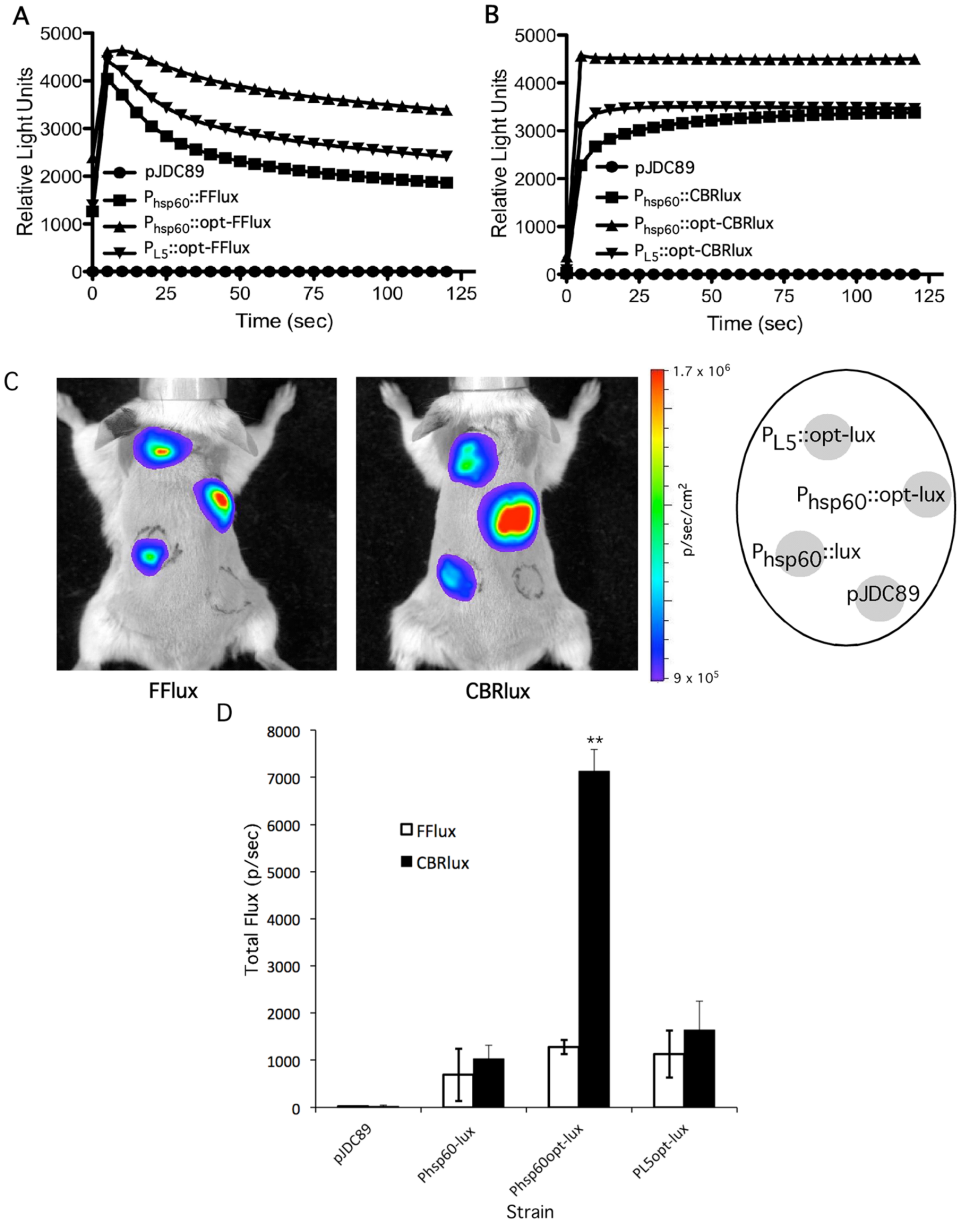
Codon optimized luciferases produce more light than non-optimized luciferases. BCG (10^4^ cfu) expressing wild type click beetle red (CBRlux) or firefly (FFlux) luciferases or the same luciferases synthetically optimized for mycobacterial codon usage (opt) were examined in liquid culture for light production over time in the presence of 2 mM luciferin (A, B). Results shown are for the individual strains of each type selected for maximal luminescence as described in the methods. BCG containing the vector alone (pJDC89) was used as a control. Both the L5 and hsp60 promoters were examined with the codon optimized luciferases to evaluate which promoter results in the greatest light production. Whole body images of BALB/C mice (2/group) subcutaneously infected with 10^6^ cfu of the same BCG strains show similar results to those found in liquid culture where codon optimized luciferases produce greater luminescence (C). A map of subcutaneous inoculation sites on mice is shown in the right panel. Quantitation of each inoculation site for total Flux photons per second (p/sec) indicates that the codon optimized CBRlux expressed from hsp60 emits the most light (D). Data and error bars represent the means and standard deviations, respectively, of two mice. ** indicates p<0.01 as compared to FFlux in the same construct.

The importance of CBRlux and FFlux for real-time analysis of mycobacterial infections makes it a high priority to further optimize their sensitivity to achieve the best thresholds of detection possible. Two different versions of each CBRlux and FFlux gene were used; one with the original DNA sequence and a second with the amino acid codon usage optimized to mycobacterial preferred amino acids (accession number JQ031640 for CBRlux and JQ031641 for FFlux). We also utilized two promoters, the mycobacteriaophage L5 promoter and the *hsp60* promoter, both of which are thought to drive high levels of transcription [42]–[45]. There was a significant increase in light production for both CBRlux and FFlux in the strains where these genes were codon optimized (Figure 7). In addition, *hsp60* promoter expression produced a stronger signal than expression from the L5 promoter. Consistent with our observation that FFlux produced higher luminescence than CBRlux in its native form, which might be due to the presence of several rare codons in CBRlux, optimization of CBRlux codon usage had a greater impact on light production than optimization of FFlux. Similar to our *in vitro* results, codon optimized CBRlux with optimized codon usage and expressed from the *hsp60* promoter resulted in higher luminescence than CBRlux expressed from the L5 promoter (Figure 7C). Overall, the optimized luciferase constructs and particularly the optimized CBRlux display significantly higher luminescence than non-optimized luciferases, suggesting that use of optimized luciferases can improve thresholds of detection beyond that observed with native luciferases.

### CBRlux allows rapid evaluation of therapeutic efficacy

**Figure 8:**
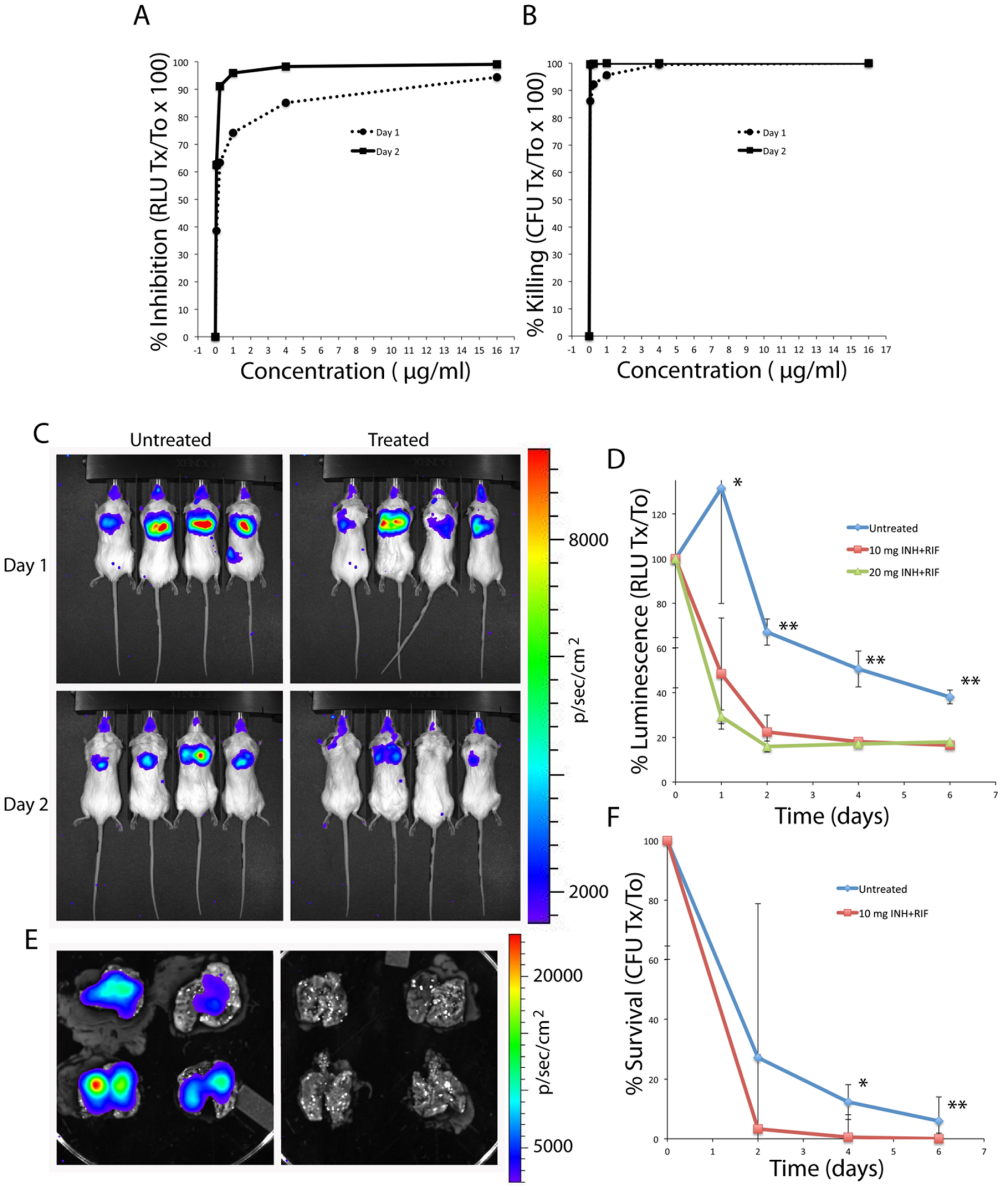
Codon optimized click beetle red luciferase (CBRlux) allows rapid therapeutic evaluation. Percent inhibition of light production (A) and bacterial killing (B) for 10^4^ of BCG expressing codon optimized CBRlux (optCBRlux) in the presence of different concentrations of isoniazid (INH) plus rifampin (RIF) during culture in bacterial media after 24 (day 1) or 48 h (day 2) as compared to the absence of antimicrobials. Whole body imaging during pulmonary infection in BALB/C mice (4/group) with 4×10^6^ cfu of BCG expressing optCBRlux after 24 and 48 hr treatment with 10 mg/kg INH+RIF results in a reduction in luminescence (C). Quantitation of the percentage of the initial luminescence as compared to each time point out to six days post-treatment confirms the reduced luminescence in treated animals (D). Similarly, ex-vivo imaging of lungs at 2 days to confirm luminescence observed in whole body images is derived from the lungs and is reduced after antibiotic treatment (E). Untreated and treated panels for lung images are indicated in panel C. Correlation of luminescence with cfu present was confirmed by plating homogenates from the same animals at each time point (F). Data and error bars represent the means and standard deviations, respectively, of four mice. * indicates p<0.05 and ** indicates p<0.01 as compared to treated group at the same time point.

Since coleopteran luciferases require ATP for the production of light, antimicrobial treatment of mice infected with luminescent mycobacteria, resulting in reduced metabolic activity or death of the bacteria, should result in a decrease in bioluminescence. Having a rapid readout for effectiveness of candidate antimicrobials would greatly facilitate development of therapeutics against mycobacterial infections, since obtaining results normally takes over a month due to the time necessary for formation of colonies on media to demonstrate a decrease in bacterial load. The ability of antibiotics to reduce signal in CBRlux expressing mycobacteria was examined by treating 10^4^ BCG in culture medium with INH+RIF for two days and comparing the inhibition of luminescence to loss of cfu (Figure 8A, B). We found that inhibition of luminescence was very similar to the level of killing observed by cfu assays, though the percent was somewhat greater with cfu, suggesting that luminescence may be a more sensitive measure of viable bacteria persisting in a population than cfu. We evaluated the utility of CBRlux for evaluating therapeutics by infecting mice with 10^6^ cfu of BCG via the intratracheal route followed by treatment with INH+RIF for six days post-infection (Figure 8C–F). We found that luminescence was significantly lower in treated than untreated animals from 24 h post-treatment. Since BCG does not replicate in mice, the bacterial load decreased throughout the experiment, as expected. However, the treated group displayed lower luminescence at all time points and lower cfu at most time points. Similar to observations in vitro, reductions in cfu appeared to be more dramatic than luminescence, but the remaining luminescence is likely due to the presence of bacteria that are not killed by treatment. These observations demonstrate that CBRlux can be used to evaluate therapeutic efficacy more rapidly than conventional cfu-based assays both in vitro and during mycobacterial infections in mice, suggesting that this reporter may be a more sensitive measure of only partial sterilization than cfu in therapeutic efficacy studies.

## Discussion

Despite decades of study into TB and *M. tuberculosis*, little is known regarding many aspects of mycobacterial disease progression and dissemination. Dissemination from the lung to pulmonary lymph nodes and other organs is thought to be required for the establishment of acquired immunity [55] but whether mycobacteria disseminate within host cells or extracellularly remains controversial. Traditional methods for quantification of bacterial burden in different organs requires sacrificing animals and plating tissue homogenates for cfu determination, which is both time consuming and costly due to the large number of animals required and the one month or more required for colonies to form on media. A growing body of literature suggests that photonic imaging can now be utilized to estimate mycobacterial burdens during infections in animals more rapidly [6], [7], [9], [10], [14], [17]–[19], [25], [26], [30], [56]. We have used FFlux and CBRlux to label pathogenic mycobacteria and visualize infection in live animals. Although the current study utilized BCG, the same vectors can be used in *M. tuberculosis*, without any expected change in signal produced, since these organisms are part of the tuberculosis-complex and very closely related [57]–[59]. This strategy enables us to follow the infection in individual mice throughout the entire experiment, reducing the number of animals needed to conduct statistically significant experiments. Each animal serves as its own control, since each individual can be followed throughout the entire experiment, reducing the experimental variability usually due to animal-to-animal differences. Theoretically, imaging could ultimately obviate the need to sacrifice animals unless localization to specific tissues must be confirmed, cellular level analyses are needed or interpretation is complicated by a change in the correlation between cfu and signal. Animals can be monitored over time with imaging and only sacrificed at key time points for more detailed analyses.

We took advantage of existing luminescent markers, CBRlux and FFlux, to label BCG by expressing the luciferases from plasmids. The two promoters used in this study show little difference in luminescence but we were able to greatly increase the signal by optimizing the codon usage in the CBRlux and FFlux genes for that preferred by mycobacteria, similar to codon optimization of luciferases described in other studies [17], [18]. Expression of either luciferase does not appear to cause growth defects in these bacteria and signal production remains at a relatively constant level up to seven days in the absence of the selective marker for the expression plasmid, even when bacteria are grown in a macrophage culture. These findings show that our constructs offer robust expression systems for CBRlux and FFlux and they have little or no detrimental effect on mycobacterial growth. Interestingly, background luminescence is extremely low with bacteria containing vector alone, but in both macrophages and mouse tissues, background with vector alone is higher, decreasing the signal-to-noise ratios and, thereby, increasing the number of bacteria required to obtain significant signal, in the presence of mammalian cells. Despite this issue, we can detect as few as 10^3^ cfu during mouse subcutaneous infection significantly above background, a very promising threshold of detection. Theoretically, these constructs should be expressed well in any mycobacterial species, including different mutant backgrounds, offering a simple method to label mycobacteria for use of *in vivo* imaging in pathogenesis, as well as vaccine and therapeutic studies.

Two different luminescence reporters, CBRlux and FFlux, with different emission spectra, allow dual bioluminescent labeling to study mycobacterial infections *in vivo*, similar to the system described for Escherichia coli [60]. The luminescence from BCG expressing FFlux or CBRlux can be localized to the site of infection with detection limits as low as 10^3^ bacteria by subcutaneous inoculation. Thus, the lower limits of detection using bioluminescence are lower than those found for *in vivo* imaging of mycobacteria using fluorescent markers [6]. This improvement in limits of detection reduces the number of bacteria required for detection, thus facilitating the use of more clinically relevant infectious doses, which is particularly important for infections with *M. tuberculosis*. However, since subcutaneous inoculations were used to determine this threshold of detection, it is likely that pulmonary infections with *M. tuberculosis* will require somewhat higher inocula to produce similar signal due to attenuation from increased mammalian tissue depth. Overall, CBRlux and FFlux, with emission maxima in the far red window, offer less signal loss due to light absorption and scattering in tissue, making them more compatible with *in vivo* imaging [39]. Furthermore, because these two luciferases use the same luciferin substrate, they can be imaged and discriminated simultaneously. We are able separate the two signals spectrally, allowing the strains that express them to be tracked and quantified. These luciferases will allow us to perform competition assays between mutant and wild type strains in the same animal. Dual color luminescence has been described with red and a green luciferases that can be spectrally separated in other systems [60], [61], but have not been used in mycobacteria. Our study supports the potential for multiple luciferases to be imaged simultaneously *in vitro* and *in vivo* using unmixing algorithms. A study dissecting the role of the adaptive immune response described the use *luxAB* labelled BCG in a RAG-2^−/−^/γ_c_R^−/−^ mouse background [30]. In this study, wild type CD90^+^ T-cells were used to complement the mutant mice and reduce luminescence to undetectable levels by 10 weeks. Our dual luciferase system could be used to analyze immunological events during bacterial infection in real time by labeling a subset of immune cells in conjunction with bioluminescent bacteria. This strategy will allow us to dissect both the bacterial and host pathways involved in mycobacterial pathogenesis. Analysis of multiple mycobacterial strains or both the pathogen and host in the same animal are only possible using multiple luminescent reporters that can be spectrally separated. It is important to remember that, due to the overlap in the wavelengths of detection for CBRlux and FFlux, it may be challenging to always accurately and sensitively discriminate them, particularly when signal levels are extremely low for one or the other of these luciferases. Further studies are needed to identify additional luciferases that may be used in this manner, possibly with more easily separated spectral characteristics, allowing analysis of at least three strains or multiple host elements during infection.

We used our luminescent mycobacterial strains to determine the effect of antibiotic treatment on light production both *in vitro* and during infection. Similar to previous studies [12]–[16], [18], [27], [31], [36]–[38], the level of bioluminescence is sensitive to increased amounts of antibiotics, suggesting that our constructs could be used to rapidly screen for efficacy of new antibiotics in high-throughput studies. Also, using *in vivo* imaging for infections and antibiotic treatment, we can verify the efficacy of antimicrobials that are effective *in vitro*, consistent with studies from other groups [38]. One caveat of the current study is that plasmids were used, which, although we observed a good correlation with cfu for seven days in the absence of selection, may not allow longer-term studies in vivo due to plasmid loss. This issue could be overcome through the use of integrating plasmids that are much more stable or placement of the constructs into specific sites in the chromosome by allelic exchange. Despite these caveats, these data demonstrate that luciferases can be exceedingly valuable for analysis of novel prevention and treatment strategies for mycobacterial infections.

We found that CBRlux and FFlux serve as highly sensitive luminescent reporters for mycobacteria, allowing determination of spatio-temporal distribution of bacteria within mice during infection. CBRlux and FFlux signals can be spectrally separated for simultaneous quantitative analysis of two different bacterial strains or bacteria along with a host cell type or marker. These luciferases can be used to sensitively detect of bacteria in pulmonary infections and can be used to evaluate therapeutic efficacy in real time. Moreover, this approach provides a novel strategy to facilitate dissecting mycobacterial pathogenesis, as well as improved understanding of therapeutic and vaccine studies.
